# Red blood cell distribution width predicts gastrointestinal bleeding after coronary artery bypass grafting

**DOI:** 10.1186/s12872-022-02875-4

**Published:** 2022-10-06

**Authors:** Ying Liao, Rongting Zhang, Shanshan Shi, Xueqin Lin, Yani Wang, Yun Wang, Weihua Chen, Yukun Zhao, Kunming Bao, Kaijun Zhang, Liling Chen, Yong Fang

**Affiliations:** 1 Longyan First Affiliated Hospital of Fujian Medical University, Longyan, 364000 China; 2 The Graduate School of Clinical Medicine, Fujian Medical University, Fuzhou, 350000 China

**Keywords:** Red blood cell distribution width, Gastrointestinal bleeding, Coronary artery bypass grafting

## Abstract

**Background:**

Red blood cell distribution width (RDW) is highly associated with adverse clinical outcomes in many diseases. The present study aimed to evaluate the relationship between RDW and gastrointestinal bleeding (GIB) after isolated coronary artery bypass grafting (CABG).

**Methods:**

This was a retrospective observational study that included 4473 patients who received CABG, and all the data were extracted from the Medical Information Mart for Intensive Care III database. Data collected included patient demographics, associated comorbid illnesses, laboratory parameters, and medications. The receiver operating characteristic (ROC) curve was used to determine the best cutoff value of RDW for the diagnosis of GIB. Multivariable logistic regression analysis was used to analyze the relationship between RDW and GIB.

**Results:**

The incidence of GIB in patients receiving CABG was 1.1%. Quartile analyses showed a significant increase in GIB incidence at the fourth RDW quartile (> 14.3%; *P* < 0.001). The ROC curve analysis revealed that an RDW level > 14.1% measured on admission had 59.6% sensitivity and 69.4% specificity in predicting GIB after CABG. After adjustment for confounders, high RDW was still associated with an increased risk of GIB in patients with CABG (odds ratio = 2.83, 95% confidence interval 1.46–5.51, *P* = 0.002).

**Conclusions:**

Our study indicates that the elevated RDW level is associated with an increased risk of GIB after CABG, and it can be an independent predictor of GIB. The introduction of RDW to study GIB enriches the diagnosis method of GIB and ensures the rapid and accurate diagnosis of GIB.

## Introduction

Heart disease is a leading cause of death and hospitalization worldwide [1]. Although many patients are treated with catheter-based interventions for ischemic heart disease, surgical approaches are still widely used [2]. Presently, approximately 370,000 coronary artery bypass grafting (CABG) surgeries are performed annually in the United States [3]. Moreover, in the United States, gastrointestinal bleeding (GIB) is a major healthcare burden that accounts for more than 500,000 hospitalizations, nearly 11 thousand deaths, and approximately five billion dollars in direct annual expenditure [4]. A non-negligible proportion of patients who experience GIB are hospitalized with other primary acute cardiovascular illnesses such as myocardial infarction, stroke, or pulmonary embolism, and GIB imposes an excess risk of short-term mortality in these patients [5]. The incidence of GIB after CABG is approximately 1.2%, but the fatality rate is high. The main reason for the high mortality rate is that early diagnosis is difficult, and some signs of GIB are easily masked by sedatives and ventilator-assisted breathing in the early postoperative period, resulting in delayed diagnosis [6].

Given the deleterious association between GIB and CABG outcome, identifying modifiable variables that can predict the occurrence of GIB after CABG and/or those that can improve their outcomes is warranted. Red blood cell distribution width (RDW) is used as a parameter to assess the heterogeneity of peripheral blood erythrocyte volume because its measurement is simple, rapid, and readily available [7, 8]. RDW can be measured with a hematology analyzer and has been used almost exclusively in the differential diagnosis of anemia for many years [9, 10]. With an increased interest in RDW in the last decade, several studies have found that RDW is a promising prognostic laboratory variable because it is an inexpensive, widely available [11, 12], and independent marker of major post percutaneous coronary intervention (PCI) bleeding [13]. Similar to patients with PCI, CABG patients are commonly treated with antithrombotics and require effective measures to reduce perioperative bleeding [14]. Studies have also shown that RDW may play an essential role in the diagnosis and management of GIB [15].

However, it remains to be determined whether RDW is an independent predictor of GIB in patients who undergo CABG. The present study aimed to investigate the relationship between RDW and GIB after CABG and to evaluate whether RDW could be considered as an additional predictive factor in the widely accepted model for predicting major bleeding risk, the CRUSADE (Can Rapid Risk Stratification of Unstable Angina Patients Suppress Adverse Outcomes With Early Implementation of the ACC/AHA [American College of Cardiology/American Heart Association] Guidelines) score [16].

## Materials and methods

### Study population

This was a retrospective study based on a publicly available Medical Information Mart for Intensive Care III (MIMIC-III) database [17]. MIMIC-III is a large, single-center database maintained by the Laboratory of Computational Physiology at the Massachusetts Institute of Technology (MIT). This database comprises information related to patients admitted to critical care units at a large tertiary care hospital located in Boston, Massachusetts, United States. This database can be accessed by passing an examination and obtaining the relevant certification. One author (YL) obtained permission to access the dataset (Record ID 36132841) and was responsible for data extraction. The use of the MIMIC-III database was approved by the review committee of MIT and Beth Israel Deaconess Medical Center (BIDMC) and was granted a waiver of informed consent.

We included 4473 patients who underwent isolated CABG and divided them into four groups according to the quartiles of RDW recorded on the first day of intensive care unit (ICU) stay.

### Variable extraction

The primary outcome of this study was in-hospital GIB. Baseline characteristics within the first 24 h after ICU admission were extracted from the MIMIC-III database, including sex, age, ethnicity, and body mass index. Details regarding the CRUSADE risk score and vital signs were also extracted. If a variable was recorded more than once in the first 24 h, we used its average value. Comorbidities identified based on the documented ICD-9 codes included coronary heart disease (CHD), congestive heart failure (CHF), hypertension, atrial fibrillation (AF), dyslipidemia, diabetes mellitus (DM), chronic kidney disease (CKD), sepsis, coagulopathy, and anemia. Acute kidney injury (AKI) was defined according to Kidney Disease: Improving Global Outcomes (KDIGO) guidelines as an increase in the serum creatinine (Scr) level by ≥ 0.3 mg/dL from the baseline within 48 h [18]. The results of routine blood tests, blood biochemistry analyses, and laboratory indicators related to glucose and lipid metabolism were collected for analysis.

### Statistical analysis

The study population was divided into four groups according to the quartiles of RDW recorded on the first day of ICU stay. Data are presented as mean with standard deviation or median with interquartile range for continuous variables and quantity and frequency (%) for categorical variables. Continuous variables were compared by one-way analysis of variance, and categorical variables were compared using the Kruskal–Wallis Rank Sum Test. The effect of RDW on the primary outcome was identified by constructing a receiver operating characteristic (ROC) curve, and the cutoff value, that is, the value that provided the best sensitivity and specificity, was determined. To assess whether the accuracy of predicting adverse outcome events could be improved by adding RDW to the widely accepted model for predicting major bleeding risk, the CRUSADE scale, the area under the curve (AUC) was calculated. DeLong’s test was used to compare the AUC of the models. The independent association between RDW (independent variable) and GIB (dependent variable) was evaluated using multivariable logistic regression models [odds ratio (OR) and 95% confidence interval (CI)]. Furthermore, the restricted cubic spline (RCS) regression model with assumed three knots was used to outline the relations between RDW and OR.

Statistical analyses were performed using the R software (version 4.0.4; R Foundation for Statistical Computing, Vienna, Austria) and SPSS statistical software (IBM SPSS Statistics, Version 24.0; Armonk, NY, USA). A two-sided *P*-value of < 0.05 was considered to be statistically significant for all analyses.

## Results

### Patient characteristics

Baseline characteristics of the patients grouped according to RDW quartiles are shown in Table 1. The study population consisted of 4473 patients with CABG. The mean age of the enrolled patients was 69.12 ± 20.61 years, and most patients were males (74.9%). The average RDW value of all enrolled patients was 13.83 ± 1.29. Patients with higher RDW values were in the elderly age group, were more frequently males, and had a higher prevalence of comorbidities, including CHD, CHF, hypertension, AF, DM, AKI, sepsis, coagulopathy, and anemia. These patients also had worse CRUSADE risk score. As expected, patients with higher RDW had lower hemoglobin and high-density lipoprotein levels and higher Scr and blood urea nitrogen levels; these patients were also more commonly receiving treatment with warfarin, heparin, and proton pump inhibitor. No significant differences were observed in aspirin, clopidogrel, tirofiban, and octreotide intake among the four groups.

**Table 1:** Baseline demographic and clinical parameters according to RDW quartiles

Categories	Overall	Q1	Q2	Q3	Q4	*P*-value
	(N = 4473)	(N = 1153)	(N = 1226)	(N = 1075)	(N = 1019)	
Age, years, mean (SD)	69.12 (20.61)	66.75 (19.98)	67.75 (16.85)	70.01 (21.36)	72.50 (23.87)	< 0.001
Male, n (%)	3350 (74.9)	937 (81.3)	965 (78.7)	786 (73.1)	662 (65.0)	< 0.001
*Ethnicity, n (%)*						< 0.001
White	1121 (25.1)	345 (29.9)	309 (25.2)	242 (22.5)	225 (22.1)	
Black	87 (1.9)	32 (2.8)	29 (2.4)	19 (1.8)	7 (0.7)	
Asian	108 (2.4)	19 (1.6)	27 (2.2)	33 (3.1)	29 (2.8)	
Hispanic/Latino	99 (2.2)	29 (2.5)	30 (2.4)	19 (1.8)	21 (2.1)	
Other	3058 (68.4)	728 (63.1)	831 (67.8)	762 (70.9)	737 (72.3)	
BMI, kg/m^2^, mean (SD)	30.15 (90.37)	27.49 (4.67)	34.10 (172.68)	29.22 (5.79)	29.40 (6.41)	0.333
HR, bmp, mean (SD)	84.93 (9.93)	84.90 (9.94)	84.86 (10.11)	84.93 (9.80)	85.04 (9.83)	0.977
SBP, mmHg, mean (SD)	112.78 (9.51)	111.96 (8.95)	112.94 (9.85)	113.01 (9.62)	113.28 (9.53)	0.006
DBP, mmHg, mean (SD)	56.74 (6.50)	56.94 (6.13)	57.23 (6.46)	56.89 (6.61)	55.75 (6.72)	< 0.001
SpO_2_, %, mean (SD)	98.00 (1.41)	98.08 (1.44)	97.97 (1.40)	97.94 (1.27)	98.00 (1.52)	0.125
CRUSADE	40.03 (14.36)	36.15 (12.86)	37.59 (13.60)	40.08 (13.84)	47.30 (14.73)	< 0.001
*Comorbidities*						
CHD, n (%)	4451 (99.5)	1150 (99.7)	1221 (99.6)	1067 (99.3)	1013 (99.4)	0.386
AMI, n (%)	1092 (24.4)	271 (23.5)	292 (23.8)	278 (25.9)	251 (24.6)	0.573
PCI, n (%)	1912 (42.7)	558 (48.4)	531 (43.3)	430 (40.0)	393 (38.6)	< 0.001
CHF, n (%)	1080 (24.1)	166 (14.4)	230 (18.8)	258 (24.0)	426 (41.8)	< 0.001
Hypertension, n (%)	3005 (67.2)	791 (68.6)	883 (72.0)	752 (70.0)	579 (56.8)	< 0.001
AF, n (%)	1718 (38.4)	369 (32.0)	429 (35.0)	433 (40.3)	487 (47.8)	< 0.001
Dyslipidemia, n (%)	1483 (33.2)	352 (30.5)	416 (33.9)	375 (34.9)	340 (33.4)	0.145
DM, n (%)	1712 (38.3)	362 (31.4)	452 (36.9)	417 (38.8)	481 (47.2)	< 0.001
AKI^a^, n (%)	3505 (78.4)	849 (73.6)	952 (77.7)	839 (78.0)	865 (84.9)	< 0.001
CKD, n (%)	420 (9.4)	39 (3.4)	84 (6.9)	87 (8.1)	210 (20.6)	< 0.001
Sepsis, n (%)	19 (0.4)	0 (0.0)	2 (0.2)	4 (0.4)	13 (1.3)	< 0.001
GIB, n (%)	47 (1.1)	9 (0.8)	3 (0.2)	15 (1.4)	20 (2.0)	< 0.001
Coagulopathy, n (%)	279 (6.2)	45 (3.9)	72 (5.9)	67 (6.2)	95 (9.3)	< 0.001
Anemia, n (%)	2134 (47.7)	462 (40.1)	489 (39.9)	547 (50.9)	636 (62.4)	< 0.001
*Laboratory tests*						
WBC, K/uL, mean (SD)	10.03 (4.56)	9.97 (4.23)	9.95 (4.19)	10.23 (4.57)	9.97 (5.30)	0.440
RBC, m/uL, mean (SD)	3.84 (0.74)	3.90 (0.72)	3.92 (0.74)	3.80 (0.74)	3.74 (0.74)	< 0.001
Platelet, K/uL, mean (SD)	208.34 (75.99)	210.56 (70.16)	206.31 (69.85)	204.90 (73.27)	211.89 (90.66)	0.098
Hemoglobin, g/dL, mean (SD)	11.71 (2.22)	12.16 (2.20)	12.06 (2.22)	11.58 (2.19)	10.93 (2.04)	< 0.001
TC, mg/dL, mean (SD)	163.68 (44.79)	170.87 (45.03)	163.98 (39.80)	163.64 (45.28)	153.16 (48.94)	< 0.001
TG, mg/dL, mean (SD)	141.61 (95.09)	135.51 (87.57)	148.27 (105.20)	139.38 (95.25)	143.07 (89.48)	0.478
LDL, mg/dL, mean (SD)	95.52 (37.75)	100.59 (37.63)	95.49 (34.21)	96.35 (37.43)	87.25 (42.13)	0.005
HDL, mg/dL, mean (SD)	43.68 (13.20)	45.60 (13.10)	43.54 (12.86)	43.66 (13.37)	41.12 (13.30)	0.008
Glucose, mg/dL, mean (SD)	137.97 (66.01)	135.17 (55.76)	138.60 (81.60)	138.98 (63.13)	139.30 (58.26)	0.416
Scr, mg/dL, mean (SD)	1.09 (0.86)	0.94 (0.40)	1.01 (0.73)	1.05 (0.72)	1.41 (1.31)	< 0.001
BUN, mg/dL, mean (SD)	20.43 (11.21)	18.06 (8.56)	18.92 (9.43)	20.18 (9.89)	25.18 (15.06)	< 0.001
Uric Acid, mg/dL, mean (SD)	6.61 (2.80)	5.67 (1.88)	5.35 (1.97)	6.66 (2.80)	7.79 (3.14)	< 0.001
RDW, %, mean (SD)	13.83 (1.29)	12.65 (0.30)	13.35 (0.17)	13.96 (0.20)	15.59 (1.47)	< 0.001
*Medications*						
Aspirin, n (%)	3811 (100.0)	913 (100.0)	1062 (100.0)	939 (100.0)	897 (100.0)	1
Clopidogrel, n (%)	1081 (28.2)	272 (29.5)	294 (27.6)	254 (26.8)	261 (28.8)	0.570
Warfarin, n (%)	1024 (26.6)	190 (20.6)	232 (21.8)	265 (28.0)	337 (37.0)	< 0.001
Heparin, n (%)	2496 (64.7)	544 (58.8)	671 (62.8)	611 (64.2)	670 (73.2)	< 0.001
Tirofiban, n (%)	15 (0.4)	6 (0.7)	6 (0.6)	1 (0.1)	2 (0.2)	0.166
Octreotide, n (%)	5 (0.1)	1 (0.1)	0 (0.0)	2 (0.2)	2 (0.2)	0.482
PPI, n (%)	1860 (48.1)	369 (39.9)	480 (44.9)	456 (47.8)	555 (60.5)	< 0.001

RDW, red cell distribution width; BMI, body mass index; HR, heart rate; bmp, beats per minute; SBP, systolic blood pressure; DBP, diastolic blood pressure; SpO_2_, pulse blood oxygen saturation; CHD, coronary heart disease; AMI, acute myocardial infarction; PCI, percutaneous coronary intervention; CHF, congestive heart failure; AF, atrial fibrillation; DM, diabetes mellitus; AKI, acute kidney injury; CKD, chronic kidney disease; GIB, gastrointestinal bleeding; WBC, white blood cell; RBC, red blood cell; TC, total cholesterol; TG, triglyceride; LDL, low-density lipoprotein; HDL, high-density lipoprotein; Scr, serum creatinine; BUN, blood urea nitrogen; PPI, proton pump inhibitor

RDW: Q1 (11.2–13.0), Q2 (13.0–13.6), Q3 (13.6–14.3), Q4 (14.3–25.7)

^a^AKI was defined according to KDIGO guidelines as an increase in serum creatinine (Scr) by ≥ 0.3 mg/dl (≥ 26.5 μmol/l) from baseline within 48 h

### RDW values and GIB

**Fig. 1:**
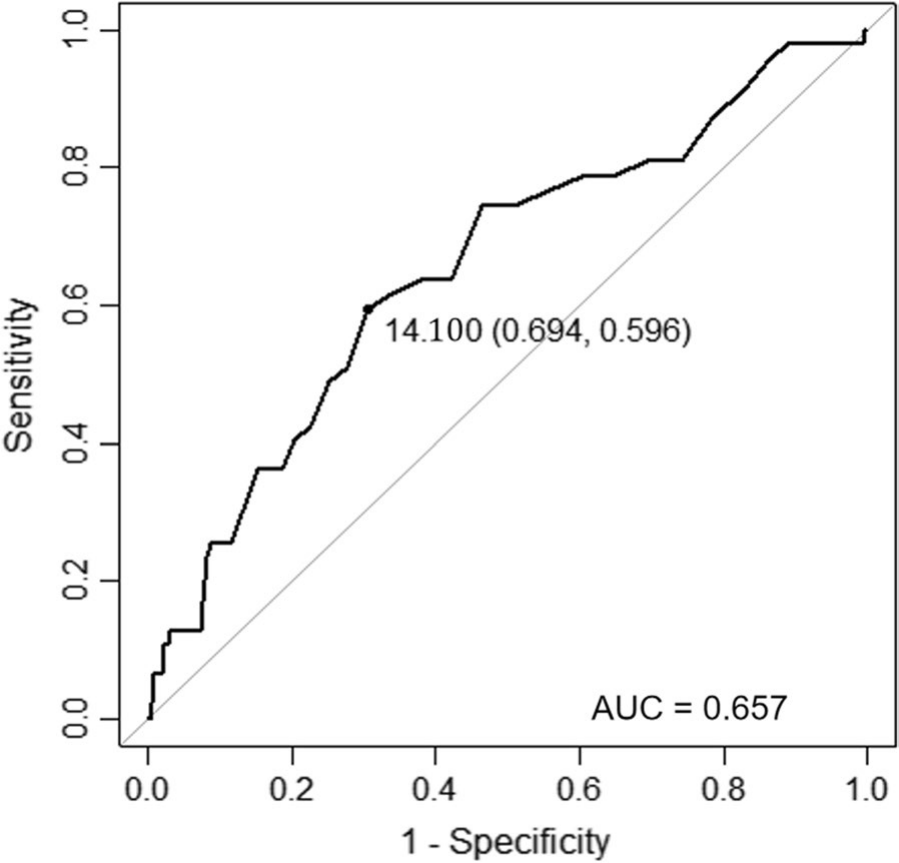
The receiver-operating characteristic (ROC) curve of RDW for predicting GIB after CABG. AUC, area under curve; RDW, red cell distribution width; GIB, gastrointestinal bleeding; CABG, coronary artery bypass grafting

**Fig. 2:**
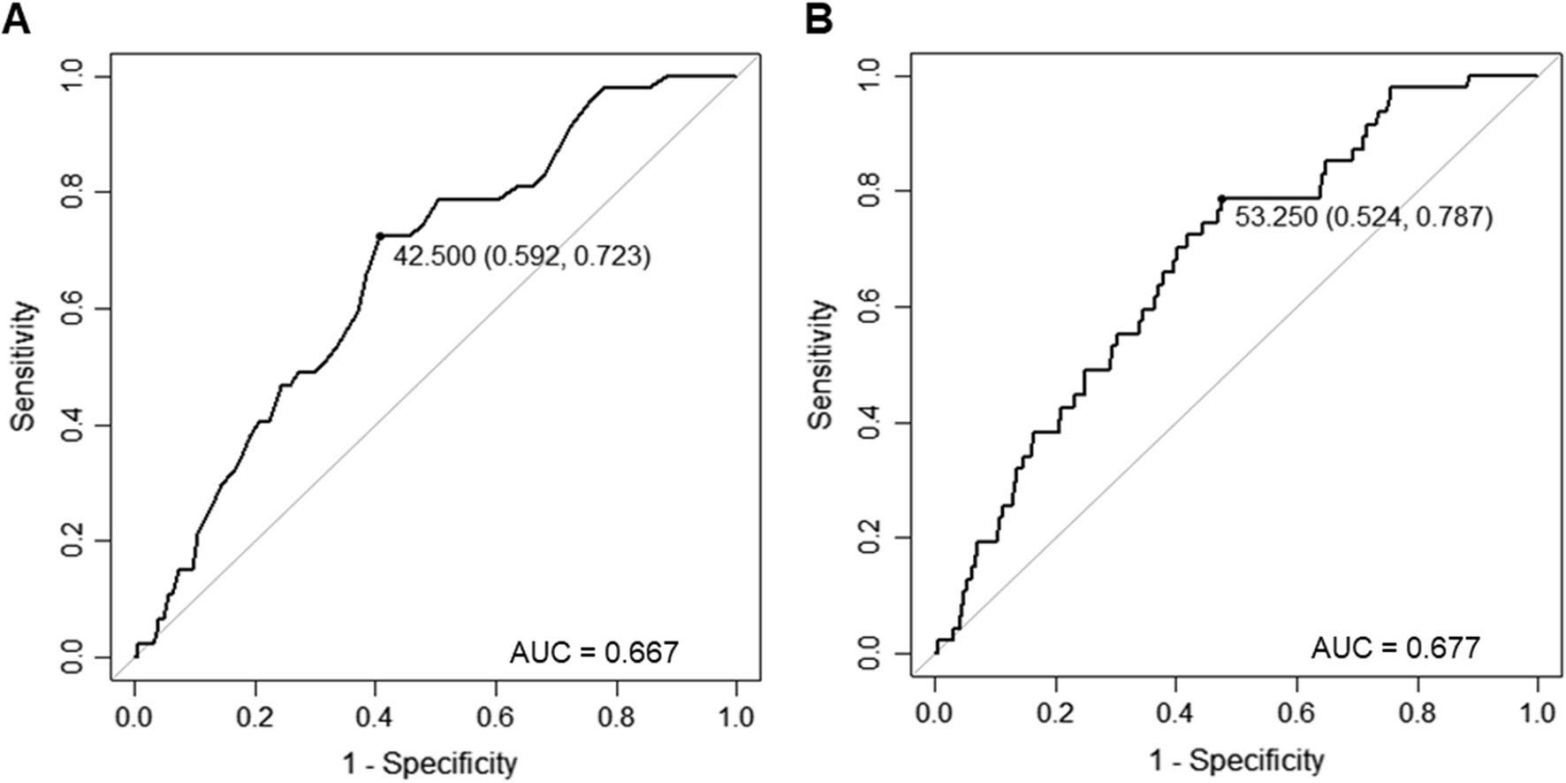
Receiver-operating characteristic (ROC) curve analysis for predicting GIB after CABG. **A** For CRUSADE risk score alone, the area under the curve (AUC) was 0.667 (95% CI 0.597–0.737); **B** When RDW was added to CRUSADE risk score, the AUC was 0.677 (95% CI 0.606–0.748, *P*-value for comparison = 0.023). RDW, red cell distribution width; GIB, gastrointestinal bleeding; CABG, coronary artery bypass grafting; CI, confidence interval

Among the 4473 enrolled patients, the AUC of the RDW for GIB was 0.657 (95% CI 0.574–0.740). The ROC curves of RDW revealed that RDW > 14.1% measured at admission had 59.6% sensitivity and 69.4% specificity in predicting GIB after CABG (Table 2, Fig. 1). The addition of RDW had a significant incremental effect on the AUC obtained from the CRUSADE risk score (AUC: CRUSADE risk score, 0.667 vs. CRUSADE risk score + RDW, 0.677, *P*-value for comparison = 0.023) (Table 2, Fig. 2A, B).

**Table 2:** Areas under the ROC Curve (AUC), sensitivity and specificity by the optimized cutoff points for RDW and CRUSADE in predicting GIB

	AUC (95% CI)	Cutoff	Sensitivity (%)	Specificity (%)
RDW	0.657 (0.574–0.740)	14.10	59.6	69.4
CRUSADE	0.667 (0.597–0.737)	42.50	72.3	59.2
CRUSADE + RDW	0.677 (0.606–0.748)	53.25	78.7	52.4

RDW, red cell distribution width; GIB, gastrointestinal bleeding

**Fig. 3:**
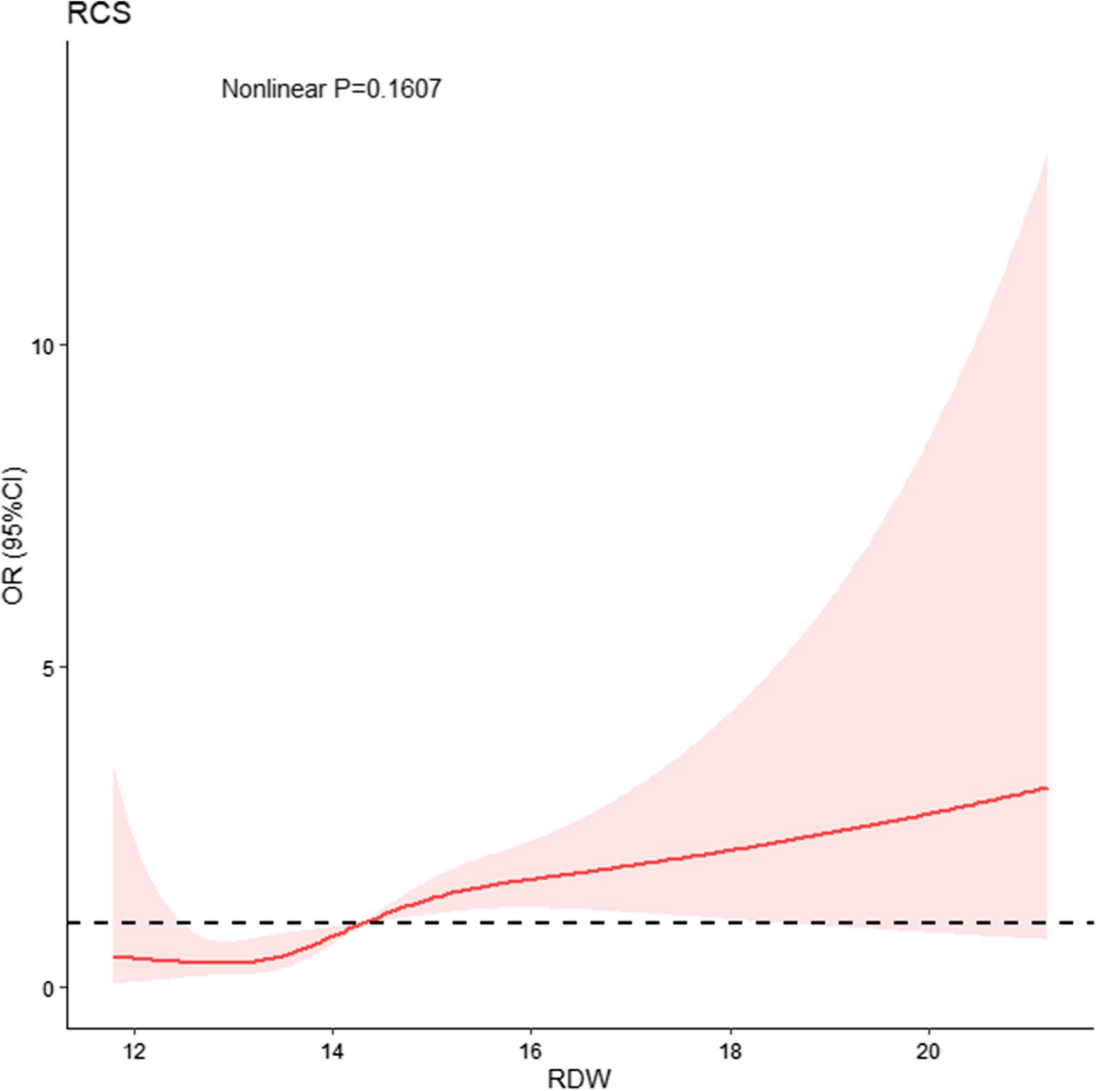
Restricted cubic spline curve for the RDW odds ratio

During hospitalization, GIB occurred in 47 (1.1%) patients. Higher levels of baseline RDW were associated with an increased risk of GIB (adjusted OR per percent increase in RDW, 1.28; 95% CI 1.09–1.47; *P* = 0.001) (Table 3). The relationship between RDW and GIB remained unchanged even after the patients were grouped into four categories according to their baseline quartile of RDW level. For example, the fully adjusted risk of GIB in the highest category of RDW was 2.83 (95% CI 1.46–5.51; *P* = 0.002) as compared to the reference group. The RCS regression model revealed that the risk of GIB increased linearly with increasing RDW (Nonlinear *P* = 0.1607) (Fig. 3).

**Table 3:** RDW in relation to gastrointestinal bleeding

Categories	Crude		Adjusted	
	OR (95% CI)	*P-*value	OR (95% CI)	*P*-value
RDW, %				
Continuous variable per %	1.32 (1.14–1.49)	< 0.001	1.28 (1.09–1.47)	0.001
Quartile^a^				
Q1–Q3	Ref		Ref	
Q4	2.54 (1.40–4.53)	0.002	2.83 (1.46–5.51)	0.002

The RDW model adjusted for aspirin, clopidogrel, wardarin, tirofiban, octreotide, heparin, proton pump inhibitor

^a^RDW: Q1 (11.2–13.0), Q2 (13.0–13.6), Q3 (13.6–14.3), Q4 (14.3–25.7)

## Discussion

To the best of our knowledge, the present study is the first to assess the relationship between RDW and GIB in ICU patients who underwent CABG. Our study provides a new biomarker for the early diagnosis of GIB. In this study, we confirmed that RDW could be used as an effective predictor of GIB in patients receiving CABG and is an independent risk predictor of GIB. Every per percent increase in RDW was positively correlated with GIB after CABG, and the fully adjusted risk of GIB in the highest category of RDW was associated with an approximately 183.0% increase in the risk in all patients as compared with the reference group. Moreover, we showed that RDW provides useful information to the CRUSADE risk score for predicting this important outcome.

It is important to identify patients who may have a better or worse prognosis. With the increased incidence of stenting of coronary lesions with a low SYNTAX score, cardiac surgeons are facing more complex coronary anatomy [19]. RDW is a quantitative measure of the variability in the size of circulating erythrocytes [20], and it has been recently found to be strongly predictive of cardiovascular outcomes in multiple patient populations, including those with acute coronary syndrome [21–25]. The study by Felker et al. published in 2007 was one of the first studies to assess the role of RDW in cardiovascular diseases, and the authors noted the usefulness of RDW as a prognostic marker in patients with heart failure [23]. Gurbuz et al. showed that baseline RDW levels were independently associated with mid- and long-term, but not with short-term, major cardiac and cardiovascular event following CABG, especially in a non-anemic population [26]. Dabbah et al. reported that RDW elevation during hospitalization after acute myocardial infarction is associated with an adverse clinical outcome [25]. A recent study also showed that RDW can be used as a predictor for saphenous vein graft disease [27].

The number of CABG surgeries in the United States is reported to be as high as 370,000 annually [3]. Patients undergoing CABG are older, and many of them have had previous myocardial infarction, stroke, or heart surgery. Consequently, morbidity and mortality after CABG surgery are expected to increase despite procedural advances. The incidence of GIB after CABG is approximately 1.2%, but the fatality rate is high [6]. Consistent with previous findings, in our study, GIB occurred in 1.1% patients. Given the deleterious association between GIB and CABG outcome, it is critical to identify risk factors that could predict GIB. Therefore, it is logical to investigate laboratory factors that could provide useful information to complement bleeding risk scales. In the present study, we found that the addition of RDW to the CRUSADE risk score improved risk stratification ability at admission.

RDW is also a valuable parameter in assessing the risk of major bleeding complications in patients undergoing PCI [13]. Our present study revealed RDW as a newly recognized and strong predictor of the risk of GIB after CABG. The mechanism through which RDW is associated with GIB, however, remains largely unknown, and this association seems to be multifactorial. Given that elevated RDW is often noted in patients with extensive comorbidities [28, 29], RDW may predict the occurrence of these diseases and other age-associated conditions. Accordingly, in the present study, patients with elevated RDW had an unfavorable baseline clinical profile that included older age, worse CRUSADE risk score, and a higher prevalence of comorbidities known to increase the risk of bleeding, such as hypertension, AF, DM, AKI, sepsis, coagulopathy, and anemia. Some studies found that patients with a higher risk of bleeding also have higher levels of C-reactive protein, suggesting that inflammation may play a role in bleeding risk [30, 31]. Other indirect mechanisms such as oxidative stress and erythropoietin deficiency and those related to iron and vitamin D3 deficiency may contribute to the increased risk of major bleeding [26, 28, 32, 33]. Further research is needed to understand the pathways through which RDW is associated with bleeding.

Our study confirmed that RDW could be used as an effective predictor in clinical practice and is an independent risk predictor of GIB in patients with CABG. The present study, however, has some limitations. First, because of the observational nature of the study, we could not establish a causal association between RDW and the risk of GIB. Therefore, our findings need to be confirmed in future studies. Moreover, although potential risk factors were adjusted for, we still cannot exclude the possibility of the existence of residual or unmeasured confounders given the observational design of the present study. Second, the data were obtained from a patient population in the United States, and thus, these results might not be completely applicable to CABG patients in other countries; the enrolled patients, however, were from different races, and therefore, the study sample has a certain level of representativeness of the global population. Third, due to database limitations, we were unable to obtain data on invasive and intensive treatment of these patients. Last, the level of RDW was assessed only once at admission. Changes in the RDW level during the follow-up period, which may have a better predictive value for adverse prognosis, were not assessed in our study.

## Conclusions

In conclusion, high RDW is a novel, important predictor of GIB in patients who undergo CABG. Importantly, CABG patients with the highest category of RDW (> 14.3%) was associated with an approximately 183.0% increased risk of GIB. Therefore, we propose that RDW could be a more simple, useful and readily available parameter for the early diagnosis of GIB. The mechanism of association requires further study.

## Data Availability

The datasets generated and analyzed during the current study are available from the corresponding author on reasonable request.

## Abbreviations

CABG: Coronary artery bypass grafting
GIB: Gastrointestinal bleeding
RDW: Red blood cell distribution width
PCI: Percutaneous coronary intervention
MIMIC-III: Medical Information Mart for Intensive Care III
MIT: Massachusetts Institute of Technology
BIDMC: Beth Israel Deaconess Medical Center
ICU: Intensive care unit
CHD: Coronary heart disease
CHF: Congestive heart failure
AF: Atrial fibrillation
DM: Diabetes mellitus
CKD: Chronic kidney disease
AKI: Acute kidney injury
KDIGO: Kidney Disease: Improving Global Outcomes
Scr: Serum creatinine
ROC: Receiver operating characteristic
AUC: Area under the curve
OR: Odds ratio
CI: Confidence interval
RCS: Restricted cubic spline
